# Health supply chain system in Uganda: current issues, structure, performance, and implications for systems strengthening

**DOI:** 10.1186/s40545-022-00412-4

**Published:** 2022-03-01

**Authors:** Eric Lugada, Henry Komakech, Irene Ochola, Shiela Mwebaze, Martin Olowo Oteba, Denis Okidi Ladwar

**Affiliations:** Management Sciences for Health, Plot 15, Princess Anne Drive, P. O. Box 71419, Bugolobi, Kampala Uganda

**Keywords:** Supply chain, Supply chain system, Health systems strengthening, Uganda

## Abstract

**Background:**

The health supply chain system is essential for the optimum performance of the healthcare system. Despite increased investments in the health supply chain system, access to quality Essential Medicines and Health Supplies remain a big challenge in Uganda. This article discusses the structure, performance, and challenges of the health supply chain system in Uganda. It provides reflections and implications for ongoing interventions for system strengthening.

**Discussions:**

The findings highlight several issues and challenges affecting the health supply chain system from functioning optimally across all levels of the health system. The challenges identified include an ineffective structure to support planning, coordination and management, inadequate funding, shortage of skilled staff, weak regulatory and governance structures at national and sub-national levels, and slow adoption and use of Electronic Logistics Information Systems to support supply chain processes and functions. Overcoming these challenges will require greater investments to improve policy development and implementation, infrastructure, equipment and support systems, knowledge and skills of supply chain personnel, increased funding and improving governance and accountability.

## Background

Access to quality essential health services and safe, effective, quality and affordable essential medicines and vaccines for all is essential for promoting health and achieving the Sustainable Development Goals (SGDs) [54]. Availability and affordability of Essential Medicines and Health Supplies (EMHS) are essential for universal access to medicines [63]. Lack of access to EMHS can result in increased avoidable morbidity and mortality, economic losses and poverty [62]. The World Health Organization (WHO) estimates that about one-third of the world’s population lack access to quality essential medicines and diagnostics [61]. Inadequate access to quality medicines and diagnostics is a major concern in many low-and-middle income countries [16]. This undermines the effectiveness of healthcare and public confidence in the health system [64].

The structure of the health supply chain system plays an essential role in optimizing the various processes and functions across the different levels of the health system. The system comprises structures and processes that ensure sourcing of equipment, commodities and supplies, purchasing and procurement, transportation and distribution of products to the end user [22], [51]. The interactions between the structures and processes have several implications on the availability of medical products across all levels of care. The key aspects that enable access to essential medicines and commodities across the health system include (a) availability, (b) affordability, (c) accessibility (geographical), (d) acceptability (rational selection and use) and (e) quality [64]. Therefore, access to EMHS is contingent on a well-functioning supply chain system that moves medicines and supplies from the manufacturer to end user health facilities. However, efficiency and effectiveness of the supply chain system is affected by several challenges including limited resources, growing complexity, inadequate inventory data, ineffective and vertical structures including inappropriate distribution to the last mile, defective forecasting and ordering issues and inadequate innovations [2].

Over the last decades, several interventions have been made with substantial investments to improve the availability of essential medicines and health commodities in health facilities across all levels of care in Uganda. These interventions have focused on several health supply chain processes and functions including human resources, infrastructure, equipment (storage facilities, computers and vehicles), information systems, medicines management, policy development, review and implementation, improving efficiency, transparency, and fostering effective collaborations between stakeholders [28], [29], [48], [52]. Despite these efforts, health facilities across Uganda continue to face difficulties accessing EMHS. In 2018, more than 80% of Health Centers (HCs) and hospitals were stocked out of one or more tracer commodities GHSC-PSM [18], [19]. Stock-outs are a result of several supply chain bottlenecks including poor inventory management, ineffective supervision and oversight, expiries of commodities, poor storage and management of commodities, and inadequate skilled supply chain system staff.

While the government and development partners have made several interventions to ensure the supply chain system is responsive to the health system needs, few if any studies have been undertaken to examine the structure, capacity, and performance of the system in Uganda. An understanding of the structure, status, and performance of the health supply chain system is key to informing policy and practice and future interventions by government and partners. More specifically, such information is critical for understanding the factors that influence the performance of the health supply chain system.

This review examines the health supply chain system in Uganda. It identifies and discusses current issues, performance, and challenges of the supply chain system across all levels of care. An analysis of the above factors and the implications for an efficient and effective supply chain system is presented. While it does not provide a comprehensive examination of the national health supply chain system, it provides a situational analysis of how the health supply chain system is organized, its performance, and implications for improving and strengthening its functionality.

## Methodology

This study is based on a narrative, exploratory literature review of the health supply chain system in Uganda. We reviewed published and unpublished literature. Published literature were retrieved from PubMed, Scopus, and Google Scholar. Other relevant literature was obtained by screening reference lists from supply chain related articles. Initially, we used “health supply chain”, “health supply chain in Uganda” as keywords to search the published papers. Following the initial search, other search terms were used including “pharmaceutical supply chain”, “medicines supply chain in Uganda”. The relevance of each paper was assessed and considered for inclusion. All relevant research articles were downloaded, read and analyzed.

An extensive review of grey literature was conducted based on initial sources retrieved from organization websites and online libraries. Several reports relating to the health supply chain system in Uganda published by the Ministry of Health (MoH), development partners, and Non-Governmental Organizations (NGOs) were obtained, reviewed, and synthesized. The main grey sources included the MoH and United States Agency for International Development (USAID) Global Health Supply Chain. Other websites searched include leading international organizations and relevant United Nations agencies (World Health Organization (WHO), the United Nations Children’s Fund (UNICEF [55], United Nations Population Fund (UNFPA).

We conducted thematic analysis and interpretation of evidence from the literature [11]. Documents were categorized in two phases. The first phase involved a description classification of research purpose, main findings, and the articles description of the health supply chain system. All documents were read and re-read, and initial themes identified and noted by two authors. Documents were categorized using open coding to generate initial themes and categories. The second step involved thematic analysis of all documents. Word frequencies were determined and used to identify potential themes. Next, emerging themes were synthesized prior to peer verification by the authors. We compared the themes with the articles purpose and overall methodology. The identified themes were compared with the coding and themes of other documents, content, and coding. Overall, the analysis process was iterative to validate the coding by returning to the documents and making comparisons with other articles. Following the thematic analysis, themes were identified and classified according to broader domains of the various processes and functional areas of the supply chain system. We categorized the findings into various supply chain subthemes including structure, regulatory frameworks and governance, personnel, logistics management information system, measure performance, data for planning and decision-making health financing, and the private sector supply chain system.

## Main text

### The structure of the health supply chain system in Uganda

**Fig. 1:**
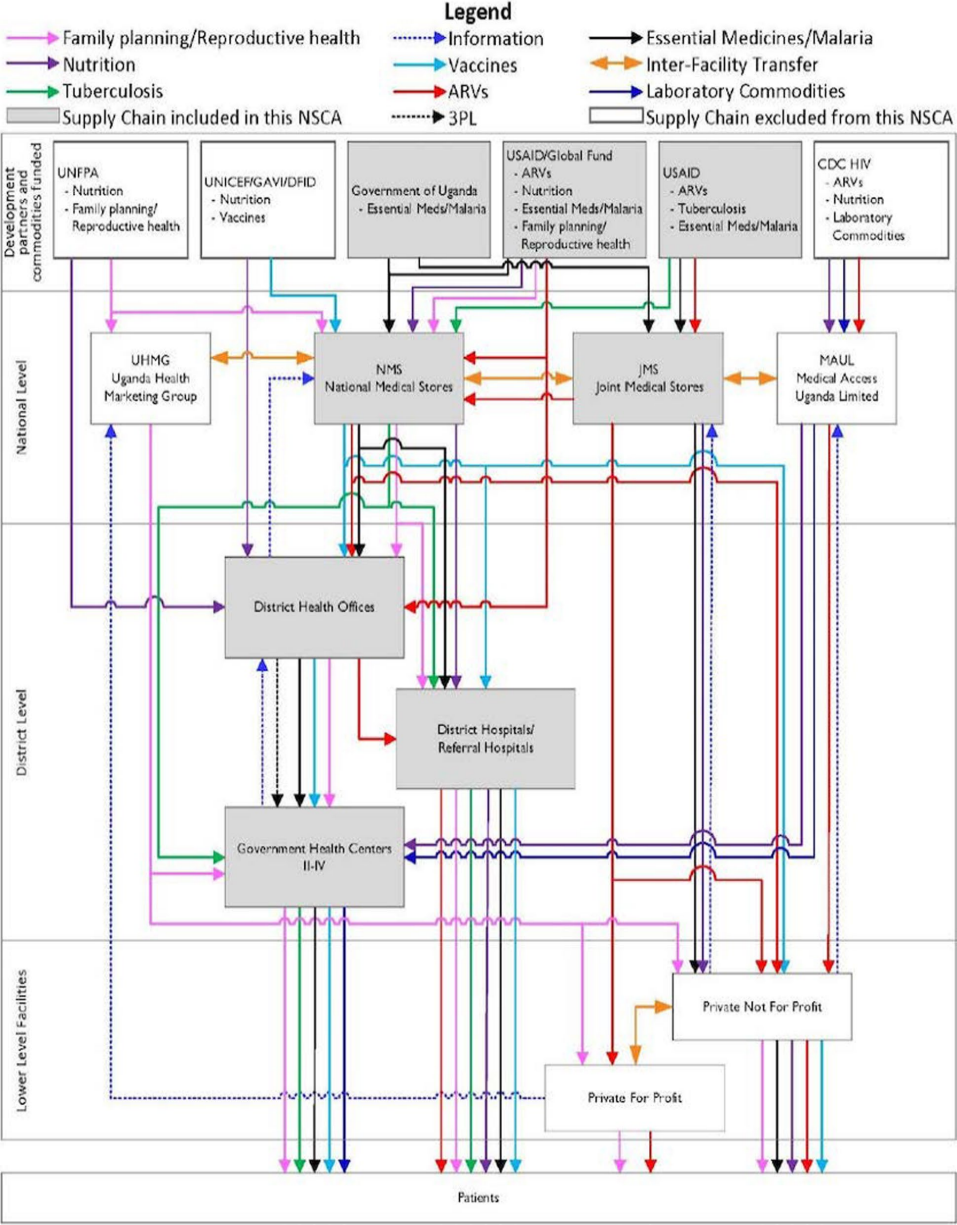
Uganda’s Supply chain structure Source: GHSC-PSM [19]. Uganda National Supply Chain Assessment Report: Capability and Performance)

The health supply chain system in Uganda is composed of both public and private stakeholders. The MoH, through the Department of Pharmaceuticals and Natural Medicines, is charged with providing oversight over policy development and implementation and coordination of the pharmaceutical sector. The National Medical Stores (NMS) and the Department of Pharmaceuticals and Natural Medicines conduct the quantification for pharmaceutical products at national level [46]. Furthermore, it directs and ensures harmonization of the supply chain system. It is actively involved in promoting the rational use of medicines and all related products in the whole country. The health supply chain system in Uganda is a four-tier system including the central level, tier II composed of sub-national level entities, tier III composed of government-managed health facilities and tier-IV composed of private health facilities (Fig. 1). The system is composed of a network of public, and private actors, development partners and Non-Governmental Organizations GHSC-PSM [18], [19].

The MoH manages the public sector health supply chain system through the Department of Pharmaceuticals and Natural Medicines. The NMS is responsible for the procurement of and distribution of pharmaceuticals and health products to all public health facilities in the country. At the district level, the District Health Office (DHO) manages the distribution of essential medicines and commodities to all facilities within its jurisdiction. The Joint Medical Stores (JMS), a Private-not-for-Profit (PNFP) institution, procures and distributes pharmaceuticals and health products to PNFP facilities in the country. Other procurement agencies include Medical Access Uganda Limited (MAUL) that is responsible for procurement, storage, and distribution of essential medicines and commodities to public, faith-based, and private health facilities. Several development partners support supply chain systems for various vertical disease programs including United Nations Children’s Fund ((UNICEF [55], Global Alliance for Vaccines and Immunization [2], Department for International Development (DFID), United Nations Population Fund (UNFPA), Danish Agency for International Development (DANIDA), The Global Fund, United States Agency for International Development (USAID) and Centre for Disease Control (CDC). Essential medicine and health commodities funded by these agencies are procured directly as an agent or on its behalf. A wide network of Private For-Profit (PFP) entities procure and distribute medical products to private health facilities and drug shops all over the country. These are composed of private distributors and wholesalers that supply medicine and health products to retail and hospital pharmacies.

### Current Issues in the health supply chain system

- **The structure of the supply chain system**
  The health supply chain system in Uganda is based on a tiered system structured around the different levels of administration in the country. The structure is designed to fit within the different administrative levels of the health system and not the technical considerations of the elements that influence the capacity and performance of the supply chain system [65]. Ensuring that there are defined structures across all levels could provide opportunities to decentralize supply chain planning, decision making and functions. However, this is yet to be effectively implemented to ensure optimum performance of the system. Failure to structure and formalize the supply chain structures has several implications for accountability for decision making and performance across the different administrative levels in the country. Furthermore, it contributes to disruptions often witnessed across several supply chain functions including forecasting, inventory management, warehouse and stores management, stock management and fails to address the commodity needs of end users (health facilities). These structural inefficiencies aggravate other supply chain challenges including insufficient coordination, inadequate financing, lack of incentivization, schemes for supply chain personnel [31], [44], [53], [66].
- **Regulatory frameworks and governance**
  Governance is a critical component of a well-functioning health system [60]. Access to quality medicines depends on strong governance, transparency and accountability, political support, and adequate financing [10]. While effective governance depends on well-established policies and frameworks, implementation is affected by several factors with several implications for the performance of the supply chain system and overall health care system [49]. Several policies and guidelines have been developed to regulate and streamline the supply chain system. However, implementation remains weak at national and sub-national levels. The MoH has made efforts to disseminate different policies and guidelines to streamline the supply chain system. However, implementation at sub national level remains weak [35], [36]**.** This affects the availability of quality essential medicines and health commodities for many people across the country. A nationwide assessment showed that less than a third, 27% of Regional Referral Hospitals and 25% of all other facilities supervised by the DHO have supply chain guidelines GHSC-PSM [18], [19]. If the guidelines are available, there was no evidence to suggest staff refer to the policies to inform decision making. Overall, weaknesses in policy formulation, review, and implementation makes the supply chain system susceptible to inefficiencies and unethical practices.
- **Health supply chain personnel**
  Human resource is an essential component of a well-functioning supply chain system [59]. Adequate, competent, and skilled workforce enables optimum performance of all other supply chain processes and functions [17]. Within the supply chain system, availability of EMHS is preconditioned on several factors including the availability of knowledgeable, skilled staff and all related support structures and systems [7]. This is critical for ensuring user units have adequate medicines and supplies that meet the health needs of individuals and households. However, the supply chain system is fraught with several human resource challenges. There is a high turnover of supply chain staff, especially at lower-level facilities. Between 10 and 55% of supply chain positions remain vacant across various levels of care. Additionally, only 6% of health facilities have integrated supply chain functions into pharmacy and stores personnel’s responsibilities. Supply chain functions integrated into pharmacy and stores personnel responsibilities include routine ordering and reporting. About 33% of health facilities have a staff recruitment policy in place GHSC-PSM [18], [19]. Other issues include inadequate training, geographical and professional isolation in hard-to-reach areas, lack of supervision/contact with supervisors, insufficient job aids, and work overload, which affect supply chain staff motivation and satisfaction [21], [23], [58]. These and other general human resource challenges continue to affect the supply chain system at all levels of care.
- **Health supply chain financing**
  Funding for the health sector has remained chronically low for several years. The health sector accounted for 5.1% of the national budget FY 2020/2021, down from 7.9% in FY 2020/2021. This is already critically low if the country is to achieve the Universal Healthcare Coverage goals. External/Overseas Development Assistance finances forms a significant proportion of the health sector resources [34]. Data on pharmaceutical expenditures are often incomplete and inaccessible compared to the overall health sector budget. However, 26% of the program nominal allocation for the FY 2020/2021 was for pharmaceutical and medical supplies (specific funding for the NMS), while 1% was for pharmaceutical and other supplies [34]. Overall, Government of Uganda (GoU) funding for EMHS increased by a total of UGX 4 billion (19,4%) from UGX 236 billion in FY 2017/2018 to UGX 275.6 billion in the FY 2019/2019 [37]. In the FY 2019/2020, only, 2.6% of the health sector budget was allocated for pharmaceuticals and other supplies. Overall, GoU funding for EMHS remains low considering the health needs in the country. This challenge is even greater since funding for EMHS by development partners focuses on specific programs and commodities covering vertical disease programs (HIV/AIDS, Tuberculosis (TB), and immunization) [33]. Funding by development partners accounts for more than 70% of public spending on medicines targeting HIV/AIDS, malaria, and TB. This is more than half of government spending on EMHS [39].
  Additionally, a large proportion of pharmaceutical expenditure is privately financed through household Out-Of-Pocket (OOP) payments. Pharmaceuticals consume a high share of total health expenditures by individuals and households. Insurance could play a significant role in supplementing the current shortfalls however, insurance and savings account for only 2.3% of all financing mechanisms in the country [40].
- **Logistic management information systems**
  Logistics Management Information Systems (LMIS) play a critical role in the performance of the different processes and functions of the supply chain system. The LMIS supports storage, transportation, waste management, forecasting, planning, and management of stock-outs. In its entirety, the LMIS ensures policymakers and service providers have access to the right data, in the right quantity and quality, at the right time, place and costs [56]. Despite the need for a well-functioning LMIS, current systems in place continue to face several challenges. The functionality of the LMIS system is affected by poor infrastructure, lack of equipment, limited internet connectivity, lack of skilled staff and/or insufficient resources for training staff across the MoH, central stores, and health facilities [25] 2014, GHSC-PSM [18], [19]. While there are several opportunities to strengthen the functionally of the supply chain system through various LMIS established by vertical programmes, collaboration remains weak between stakeholders. Most of the infrastructure, equipment and human resource capacity, have been developed for specific vertical disease projects [9].
  Digitalization is essential in improving the operations, performance, and sustainability of the supply chain system. Digitalization offers an opportunity to integrate innovative technologies, focus on client care, reduce intra and inter organizational costs and create more value for the supply chain system. This can be achieved through the implementation of data capture technologies, strengthening data quality, and increasing data use [8]. Several digital supply chain platforms have been either adopted or developed and being used to support the supply chain system in Uganda. The District Management Information Software (DHIS-2), electronic Logistics Management Information System (eELMIS) are key platforms supporting supply chain functions of the health system [38]. Several Enterprise Resource Planning (ERP) applications currently support the health supply chain system including NMS SmartCare, NMS Distribution Monitoring Tool (DMT) [47]. While digitalization has been emphasized as a key factor for improving operations and performance of the health supply chain system, several challenges continue to affect its use. The current digital health supply chain eco-system is erratic, fragmentated and disconnected between different functionalities, and levels of operations in the country [41].
- **Health supply chain performance**
  The performance of the health supply chain system is an essential factor for ensuring efficiency and effectiveness of the health system. The supply and availability of EMHS is important for health facilities to enhance performance in terms of quality, costs, responsiveness, and patient satisfaction. The GoU, through the MoH and its partners have made several efforts to improve the performance of the supply chain system and ensure access to quality EMHS. However, the performance of the health supply chain system has been sub-optimal. A national survey found that regarding warehousing and storage, less than half, 47% of hospitals had enough buffer or security stock in inventory management system. Furthermore, only 25% of lower-level facilities and 23.5% of hospitals were stocked according to plan. Data performance based on the use of the electronic Logistic Management Information Systems accuracy remains low across all levels of care for most commodities. The ELMIS data accuracy is generally low at 33% for lower-level facilities and 20% of hospitals. On average, both lower-level facilities and hospitals are stocked out for 6 days GHSC-PSM [18], [19].
- **Data to support planning and decision making**

Health systems depend on quality and timely data from the Health Information System [6] to support decision-making that is critical for overall system performance [1]. Quality and reliable data is essential to inform planning and management of the supply chain system to ensure that it is responsive to the needs of health facilities across all levels of care. Planners, and decision-makers need to know the rate at which commodities are being consumed, at each point of care to create a supply process that warrants quality services while minimizing stock-outs. However, there are several barriers to inform data-driven decision making and improvements in the health supply chain system. Several functions of the LMIS including data archiving, accurate and timely reporting and tracking of indicators, and the use of data remain a challenge. The ELMIS supports several supply chain functions. However, the system is not frequently updated as may be required (only after three years) or when there is need for adjustments to the system. A significant proportion of service delivery points in Uganda still rely on non-electronic data management systems leading to data loss, poor analysis, limiting data sharing for decision making GHSC-PSM [18], [19].

#### Performance of the supply chain system

Measuring the performance of the supply chain system is essential for ‘tracking and tracing” of efficiency and effectiveness that is key for informing decision making and policy formulation processes [3]. Performance measurement forms the basis for ensuring optimum performance of the health supply chain system. Despite several interventions there is inadequate information on the performance of the supply chain system.

The supply chain system in Uganda is complex with many actors GHSC-PSM [18], [19]. Many of these entities have predetermined goals and performance indicators and optimization criteria. Additionally, the existence of different sources procuring similar commodities using parallel supply chains complicates the harmonization of performance measurement. Such complexities do not contribute to the overall supply chain system as one entities performance improvement may have negligible effects. This challenge is further complicated by inadequate data to inform measuring the performance of the supply chain system [42]. While efforts have been made to harmonize performance indicators, lack of accuracy and consistency in measurement means there is inadequate information that can be used for benchmarking and informing decision making.

#### Private For-Profit sector supply chain system

The PFP sector plays a key role in the provision of medical products and services in Uganda. The private sector supply chain system comprises manufactures, importers, and distributors (wholesale and sub-wholesale) of a range of medical products and supplies [65]. There are an estimated 28 manufacturers, 70 importers and distributors, 597 wholesale pharmacies, 1179 retail pharmacies, 4742 authorized drug shops, and an unknown number of illegal providers [45]. The PFP sector is regulated and licensed by the NDA. Other private bodies and associations provide further guidance and regulation [20]. The PFP sector accounts for the largest proportion of medical products and supplies in the country [27]. These play essential supply chain roles between manufacturers (in-country and outside) and the end users, mostly pharmacies, drug shops, and health facilities. The PFP sector wholesalers’ distribution of medicines is based on orders and consumption by the user units (pharmacies and health facilities). Private pharmacies are predominantly located in urban areas supplying health facilities and smaller units (drug shops) with various commodities.

The availability of quality medicines is critical for promoting trust among users and improving health outcomes. A study by [30] showed that there are more 84.3% PFP provider drug outlets compared to 13.0% public providers. Many of these PFP facilities are unlicensed and unsupervised by the relevant authorities [12]. In addition, availability of EMHS is higher among PFP sector providers compared to the public facilities [4]. However, the quality of medicines procured and distributed by PFP retail outlets (pharmacies and drug shops) across the country has raised several questions. The NDA is mandated with regulation and enforcement of guidelines and standards [20]. However, weak supervision and oversight particularly at sub-national level (districts) means not all pharmacies and outlets are supplying quality medical products [5]. Retailers may not have the ability to assess the authenticity and quality of medical products they purchase and sell to end users.

Pharmacies and drug shops are required to recruit qualified pharmacists to manage the stores, wholesale, and retail outlets. However, many private pharmacies and drug shops across the country face an acute shortage of trained pharmacists. Many pharmacies employ unqualified personnel to manage the sales and distribution of medical products [24]. This could be due to several reasons including generally inadequate qualified pharmacists across the country and the preference of proprietors to employ other categories of staff (nurses and midwives) who may not cost as much in terms of payment. These challenges are greatest in peri-urban and rural areas where supervision and oversight by respective authorities in the districts may be weak [12].

### Implications for strengthening supply chain systems

Despite the issues, and challenges discussed, several opportunities exist from completed or ongoing interventions by the government and partners that can be leveraged on to strengthen the supply chain system. Strengthening the supply chain system may offer opportunities for greater health systems performance [13]. In recent years, the evidence base demonstrating what works to strengthen health supply chain systems in developing countries has been increasing [14], [15], [29], [43], [50], [57]. These studies highlight several strategies and interventions that have been used to strengthen different supply chain processes and functions including procurement and financing, systems design, supervision, training and management, the structure of the system, mobile phone-based technology, and quality improvement measures. Furthermore, the supply chain system is dynamic and diverse. Therefore, given the contextual factors that influence the effectiveness and performance, these strategies, or a combination of could guide the strengthening of the supply chain system.

#### Strengthening governance, transparency, and accountability

Effective governance and absence of corruption are key for ensuring medicines and supplies are available in health facilities across all levels of care in Uganda. This ensures that individuals and households have access to safe, effective, quality medicines through strong management, transparency and accountability of the supply chain system [26]. To ensure robust governance, policies and regulatory bodies in Uganda, it is important for the Ministry of Health and related governments institutions at all levels to take up greater ownership of the health supply chain system. This should include strengthening political support, facilitating dialogue with all health supply chain stakeholders to ensure their participation [32]. It is important to strengthen accountability mechanisms throughout the health supply chain system to ensure that all stakeholders tasked with management and performance monitoring including policy makers, health service managers, service providers are responsive to the commodity needs across all levels of care. In addition, clients or health consumer groups need to be empowered to monitor the quality of services, demand for greater accountability and better services and performance of the supply chain system across all levels of care. Finally, it is important to guarantee public access to key information about the supply chain system to strengthen accountability.

#### Strengthening supply chain personnel

Availability of adequately skilled and effectively distributed health supply chain staff across the system is critical to ensuring equitable access to quality medicines across all levels of care. The GoU and MoH needs to prioritize and develop a strategy to recruit and retain adequately skilled supply chain staff to serve the central, sub-national stores and all levels of care including Health Centers HCs (II, III and IV) and all hospitals. This should be matched with strategies to ensure that the capacities of existing staff are strengthened through continuing education/on-job training on critical supply chain practices, leadership and management and development of feedback and supervision mechanisms across all levels of the supply chain system. This could be achieved through the development and offering adaptive learning strategies including e-learning and mentorship programs. Similarly, offering training opportunities for staff to continue to grow through collegiate programs, on-the-training scheme, and classroom instructions. It is important to develop strategies to ensure that renumeration and compensation of health workers is commensurate with qualification and experience and improved in both public and private health sector supply chain systems. Additionally, there is a need to develop long-term strategies for sustained incremental funding by the government to maintain appropriate levels of supply chain personnel. This should be strengthened by devising performance incentives and recognition schemes to attract skilled talent,increase and maintain motivation among supply chain personnel.

#### Establish and strengthen supply chain coordination frameworks

Coordination plays an essential role in ensuring effective supply chain management and performance among the various stakeholders, including government ministries, departments, donors and development partners, and health facilities. The need to streamline all supply chain coordination frameworks given the existence of multiple stakeholders with different and sometimes conflicting goals and mandates. Above all, a streamlined coordination framework is critical to ensuring efficient use of available resources and optimal performance of the health supply chain system. To strengthen supply chain management, the MoH should strengthen current coordination frameworks at all levels to facilitate decision-making that is critical for ensuring effective oversight and monitoring performance of the health supply chain system. This could be accomplished by mapping supply chain coordination structures at national and sub-national levels, defining coordination needs and processes, establishing functions of the coordination frameworks and developing means of monitoring performance and ensuring accountability. This is key to ensuring that there is a mechanism to support the supply chain process, functions, and relationships through all levels of the health system.

#### Streamline and strengthen Logistic Management Information System and information flow

Management of health supply chain data and its use to support decision making remains a challenge in Uganda. Currently, there are several LMIS sub-systems functioning with limited if any form of integration. The importance of an efficient and harmonized LMIS cannot be overlooked for the health system is to become effective and responsive. As such, the supply chain system requires real time data about stock status, consumption, and other important variables. It is critical to ensure the LMIS provides comprehensive, accurate, complete, and timely data to inform actions and interventions by supply chain stakeholders. Additionally, it is important to disseminate reports regularly to different health supply chain stakeholders and ensure the free flow of feedback on performance at the different levels. This goes along with strengthening the capacity of managers and health facility staff in data management and analysis, dissemination and use to support decision making across all levels of care. Overall, to achieve end-to end supply chain data visibility, it is essential to digitize the supply chain system, standardize data across all levels of care, strengthen data security and provide incentives to promote data use across all levels of care.

The electronic Logistic Management System (ELMIS) is an innovative, cost-effective system for health logistics data management. The ELMIS ensures availability of data hence greater commodity and better health outcomes. However, the Information, Communication and Technology (ICT), infrastructure, equipment support systems, and competence are still weak limiting the functionality of the ELMIS across the health supply chain system. It is important to develop a plan that will ensure the availability of infrastructure and equipment and internet coverage across all levels of the supply chain system. The MoH should develop a timeline and roadmap to transition to ELMIS to minimize the duplicative paper based LMIS processes and functions. This should be complemented with capacity building efforts with key notable activities including developing and implementing staff (re)training on ELMIS use.

#### Measurement of supply chain performance

Performance measurement is a pre-requisite for enhancing the supply chain system performance and accountability. Given the resource constraints, the supply chain system faces new pressures for greater accountability for impact notably, the availability of quality EMHS across all levels of the healthcare system. Performance measurement systems would assist the stakeholders in decision making to improve the effectiveness and efficiency of operations and demonstrate the capacity and performance of the supply chain system. It is important to establish harmonized performance goals within and between the various supply chain functional areas that will lead to the desired results that are balanced and without disputes. The performance goals should be embedded in the fabric of supply chain service delivery and management of the functions of the system. In this way, each level of accountability will be responsible for delivering its part of the supply chain performance goals. It is important to strengthen current national and sub-national performance assessment approaches to ensure the systems are systematic, transparent, and rigorous in capturing and reporting performance of the system. Additionally, the performance measurement strategy should be as comprehensive as possible to ensure, it assesses the entire health supply chain structure, process, and outcomes.

#### Strengthening private sector supply chain

The public sector is a prominent stakeholder in ensuring availability of EMHS across all levels of care in Uganda. However, it is important to acknowledge the role of the private for-profit providers in the health supply chain system. The private sector plays a significant role in supply chain-related markets including manufacturing, importation, retailing, and delivery of health commodities. While engagement between the public and private health supply chain stakeholders remains low, the latter plays a significant role across the various functional and technical areas. These roles have often been accomplished with high levels of efficiency and effectiveness by the private for-profit providers. However, there are no frameworks to guide public–private engagements within the health supply chain system. A comprehensive public private for-profit engagement framework will provide a clear understanding of needs, opportunities, and strengths of both sectors to ensure each meets the broader supply chain goals. It is essential to engage the private for-profit providers when developing, reviewing and implementing policies to support the design of flexible mechanisms and regulations that address and streamline their roles in a rather complex supply chain environment. Further, it is important to support and strengthen private for-profit supply chain associations and groups such as importers, wholesalers, retailers, and health facilities to strengthen regulation, monitoring, enforcing quality and performance of its members.

Several limitations must be kept in mind when considering the findings and conclusions of this paper. First, the quality of the documents reviewed was not assessed as in several cases not all necessary information was provided. Secondly, while we felt that the sample of the documents reviewed is representative of the literature on the health supply chain system, there may have been some bias associated with the narrow focus of the reviews. As indicated, the review included only literature in the areas of the health supply chain system. We did not consider and excluded documents from other disciplines. Finally, since only a few peer-reviewed articles could be included after a reference check, we do not rule out the fact that all relevant studies were included.

## Conclusion

The article examined the status, performance of the supply chain system in Uganda. Like other supply chain systems, the health supply chain system plays a critical role in achieving national development and international goals. Uganda's supply chain system is complex, consisting of numerous stakeholders, and is characterized by several interactions from all sizes of entities. Health supply chain management, like in all other sectors, is applicable to the health system and offers guidelines that can be used to analyse, coordinate, and constantly improve the whole system, thereby ensuring access to EMHS across all health system levels. Through structured review and synthesis of the available evidence, several themes, namely structure of the supply chain system, supply chain performance, logistics information systems, health supply chain personnel, sustainable financing, and governance and leadership. These factors and how they affect the availability of EMHS and implications for supply chain systems strengthening.

The findings show that the capacity and performance of the supply chain system continue to improve in several technical and functional areas, evidenced by the increased availability of EMHS across all levels of care. However, the synthesis highlighted several challenges affecting the health supply chain system that needs to be addressed. Findings from the synthesis show that while gaps in the technical areas identified are being addressed, several remain pertinent. Overall, these point to the need for further investments, improvements, and interventions in the identified technical and functional areas and to strengthen the capacity and performance of the supply chain system.

## Data Availability

Not applicable.

## Abbreviations

CDC: Centre for Disease Control
DFID: Department for International Development,
DHO: District Health Officer
ELMIS: Electronic Logistics Management Information System
EMHS: Essential Medicines and Health Supplies
HC: Health Centers
MAUL: Medical Access Uganda Limited
MoH: Ministry of Health
NGOs: Non-Governmental Organizations
NMS: National Medical Stores
PNFP: Private-not-for-Profit SGDs: Sustainable Development Goals
UNFPA: United Nations Population Fund
JMS: Joint Medical Stores
USAID: United States Agency for International Development
UNHCR: United Nations High Commission for Refugees
UNICEF: United Nations Children Emergency Fund
WFP: World Food Programme
WHO: World Health Organization
